# Evolution of clitellate phaosomes from rhabdomeric photoreceptor cells of polychaetes – a study in the leech *Helobdella robusta* (Annelida, Sedentaria, Clitellata)

**DOI:** 10.1186/1742-9994-10-52

**Published:** 2013-09-05

**Authors:** Carmen Döring, Jasmin Gosda, Kristin Tessmar-Raible, Harald Hausen, Detlev Arendt, Günter Purschke

**Affiliations:** 1 Universität Osnabrück, Zoologie, Osnabrück 49069, Germany; 2 Present address: Kopernikusstrasse 5, 48477 Hörstel, Germany; 3 Max F. Perutz Laboratories, Universität Wien, Campus Vienna Biocenter, Austria Research Plattform “Marine Rhythms of Life”, Dr. Bohr–Gasse 9/4, 1030 Wien, Austria; 4 Sars International Centre for Marine Molecular Biology, Thormøhlensgate 55, 5008 Bergen, Norway; 5 Developmental Biology Programme, European Molecular Biology Laboratory, Meyerhofstraße 1, D-69012 Heidelberg, Germany

**Keywords:** Annelida, Cell type, Clitellata, Evolution, Eye, Opsin, Phaosome, Photoreceptor cell, Phylogeny

## Abstract

**Introduction:**

In Annelida two types of photoreceptor cells (PRCs) are regarded as generally present, rhabdomeric and ciliary PRCs. In certain taxa, however, an additional type of PRC may occur, the so called phaosomal PRC. Whereas the former two types of PRCs are always organized as an epithelium with their sensory processes projecting into an extracellular cavity formed by the PRCs and (pigmented) supportive cells, phaosomes are seemingly intracellular vacuoles housing the sensory processes. Phaosomal PRCs are the only type of PRC found in one major annelid group, Clitellata. Several hypotheses have been put forward explaining the evolutionary origin of the clitellate phaosomes. To elucidate the evolution of clitellate PRC and eyes the leech *Helobdella robusta*, for which a sequenced genome is available, was chosen.

**Results:**

TEM observations showed that extraocular and ocular PRCs are structurally identical. Bioinformatic analyses revealed predictions for four opsin genes, three of which could be amplified. All belong to the rhabdomeric opsin family and phylogenetic analyses showed them in a derived position within annelid opsins. Gene expression studies showed two of them expressed in the eye and in the extraocular PRCs. Polychaete eye-typic key enzymes for ommochromme and pterin shading pigments synthesis are not expressed in leech eyes.

**Conclusions:**

By comparative gene-expression studies we herein provide strong evidence that the phaosomal PRCs typical of Clitellata are derived from the rhabdomeric PRCs characteristic for polychaete adult eyes. Thus, they represent a highly derived type of PRC that evolved in the stem lineage of Clitellata rather than another, primitive type of PRC in Metazoa. Evolution of these PRCs in Clitellata is related to a loss of the primary eyes and most of their photoreceptive elements except for the rhabdomeric PRCs. Most likely this happened while changing to an endobenthic mode of life. This hypothesis of PRC evolution is in accordance with a recently published phylogeny of Annelida based on phylogenomic data. The data provide a nice example how morphologically highly divergent light sensitive structures emerged from a standard type of photoreceptor cell.

## Introduction

A functional eye requires two fundamental building blocks: photoreceptors and shading pigment [1,2]. These functions may occur together in one cell type or they are separated and exhibited in two or more different cell types [2], commonly referred to as photoreceptor cells (PRCs) and pigmented supportive cells (PSCs) (see [3], for terminology). In Metazoa generally two types of PRCs can be recognized; rhabdomeric and ciliary PRCs [1,4-6]. For photoreception these cells use either microvilli or cilia. These cell types can be distinguished not only morphologically, but also by cell-type-specific sets of molecular markers, their so-called molecular fingerprint. These characters also enable clarifying their evolutionary history and diversification [2,5,7-12].

In Annelida these two types of PRCs are considered to be generally present, but in certain taxa an additional type of PRC may occur, the so-called phaosomal PRC [13]. The former two types of PRCs are always organized as an epithelium with their sensory processes – cilia or microvilli - projecting into an extracellular cavity formed by PRCs and supportive cells. Phaosomes, however, are seemingly intracellular vacuoles housing the sensory processes which may be cilia or microvilli as well [13,14]. Phaosomal PRCs are rare in polychaetes, but are typical for Clitellata and constitute their only known type of photoreceptor cell [15,16]. In Clitellata these phaosomal PRCs primarily occur extraocular and are present within or outside the brain [16-21].

Whereas phaosomal PRCs are widespread or even ubiquitous in Clitellata, pigmented eyes occur only exceptionally and are restricted to species of Naidinae, Pristinae and Hirudinea [15,16,22]. In contrast to their soil-dwelling relatives, species of these groups are characterized by inhabiting the littoral zone of various freshwater habitats (certain leeches may even be termed terrestrial). The eyes in these taxa are not as closely related to the brain as is the case in polychaetes. Although in Naidinae they are prostomial, they may not represent cerebral eyes [16]. They are segmental in leeches, occurring from segment II onward posteriorly in one or several pairs [22-24]. These eyes are composed of PRCs and PSCs [16,17,22-24]. They exclusively employ phaosomal PRCs as well, indicating an evolutionary origin independent from that of the pigmented eyes present in polychaetes, which use rhabdomeric PRCs [13,16].

Several hypotheses have been put forward explaining the evolutionary origin of the clitellate phaosomes. These cover a wide range of scenarios from either being comparatively primitive PRCs or having newly evolved in the stem lineage of clitellates [15,16]. Whether these PRCs really represent a cell type completely evolved de novo and independently from both ciliary and rhabdomeric PRCs or whether they may have been derived from one of these cell types may only be evaluated by tracing the molecular fingerprints of phaosomal PRCs. The investigations of Ukhanov and Walz [25] on phototransduction in leech eyes give some indication for a relationship to rhabdomeric PRCs. In this study we addressed this question by investigating gene expression patterns in the phaosomes of a leech, *Helobdella robusta* Shankland et al., 1991. This species was chosen for several reasons: it is an example of a clitellate with both extraocular and ocular phaosomes, a sequenced genome is available, the species can easily be cultured in the lab, and *in situ* protocols are available. Although TEM investigations are available for another *Helobdella* species, *H. stagnalis* (Linnaeus, 1758) [17]; we complemented the ultrastructural data for the eyes in *H. robusta.* In addition we described the extraocular phaosomes and also looked for the presence of putative ciliary PRCs. The data on *opsin* expression and phylogenetic analysis of *opsin* sequences as well as the presence of a gq protein in the phaosomal PRCs are indicative for polychaete adult eye PRCs. These data provide strong evidence that the clitellate PRCs evolved from the rhabdomeric PRCs present in these eyes. Due to the absence of expression of either *tryptophane-2,3-dioxygenase* and *sepiapterine reductase* in leech pigmented supportive cells (PSCs), genes which are typical for the pigment synthesis pathway in polychaete eyes, the PSCs in leeches most likely have no counterparts in the eyes of polychaetes.

## Results

**Figure 1:**
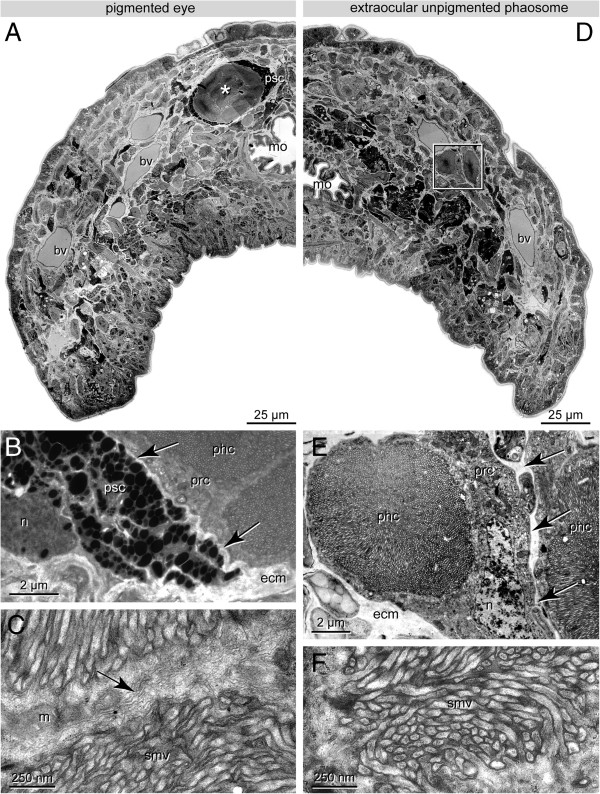
***H. robusta *TX; TEM observations of pigmented eyes (A-C) and extraocular PRCs (D-E). A**. Low power micrograph showing half of a cross section, pigmented eyes are made up of several phaosomal PRCs (asterisk) surrounded by a thin layer of pigmented supporting cells (psc). Eyes embedded in the connective tissue below the epidermis. **B**. The PRCs of the eye and the PSCs are separated by ECM (ecm, arrows), PRCs (prc) are closely apposed. **C**. Border between two adjacent PRCs (arrow); note densely arranged sensory microvilli (smv) and mitochondria (m). **D**. Low power micrograph showing half of a cross section anterior to the eyes; extraocular phaosomal PRCs (boxed) dispersed along the anterior head margin. **E**. Extraocular phaosome, sensory microvilli fill the entire phaosomal cavity (phc). The nucleus (n) is at one edge of the PRC (prc) forming the phaosomal cavity. Note ECM separating two adjacent PRCs (arrows). Detail of D. **F**. Enlargement of sensory microvilli (smv) cut in various directions. Note dense filamentous core in each microvillus. - asterisks: pigmented eyes; bv blood vessel; ecm ECM; m mitochondrion; mo mouth opening; n nucleus; phc phaosomal cavity; prc photoreceptor cell; psc pigmented supportive cell; smv sensory microvilli.

**Figure 2:**
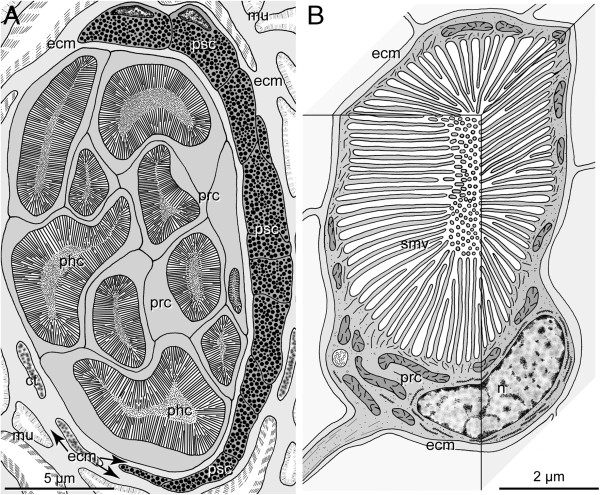
***H. robusta *TX; schematic representation of TEM observations of pigmented eyes and PRCs. A**. Section through an eye at the level of the phaosomes. The eyes are embedded in the connective tissue (ct) which primarily comprises muscle fibres (mu). Ten PRCs (prc) with their phaosomal cavities (phc) are visible, pigmented supportive cells (psc) form a thin sheath around the PRCs; the two cell types are separated by an ECM (ecm, arrows). **B**. 3-D view of an extraocular PRC (prc) with its phaosome and sensory microvilli (smv), PRC completely ensheathed by ECM (ecm). - ecm ECM, mu muscle fiber, n nucleus, phc phaosomal cavity, prc photoreceptor cell, psc pigmented supportive cell, smv sensory microvilli.

In *H. robusta* only one pair of pigmented eyes is present. It is situated in the region of the anterior sucker on the third annulus and deeply embedded in the connective tissue below the epidermis (Figures 1A and 2A); in the juvenile specimens studied, this area is about 50 μm behind the anterior tip of the animals.

### Electron microscopic observations

In *H. robusta* the eyes generally have the same structure as described by Clark [17] for *Helobdella stagnalis* (Linnaeus, 1758). The eyes are of the pigment cup type [3] opening to left anterior in the left eye and to right anterior in the right eye (Figures 1A and 2A). In the juvenile specimens investigated each eye comprises 10–15 phaosomal PRCs covering an area of about 8 by 16 μm on cross sections. In the pigmented eye the PRCs are close together and not separated by ECM (Figures 1C and 2A). The cell bodies of the PRCs are located at the opening of the eye cup. The phaosomes are somewhat staggered in the eye cup so that usually no more than 10 phaosomes are visible on a given section. The phaosomes are completely occupied by densely packed sensory microvilli, leaving no central space inside the cavity (Figure 1B).

The pigmented supportive cells (PSCs) form a comparatively thin sheath of about 0.7-1.5 μm around the PRCs, providing space for at least 2–7 layers of membrane-bounded pigment granules. At certain areas there is some overlap of pigment cells (Figure 1A, B). The PSCs border directly on one another but are separated by an ECM (0.1 μm wide) from the PRCs (Figures 1B and 2A). Their nuclei are situated on the convex side of the pigment cup (Figures 1B and 2A).

Several extraocular phaosomal PRCs occur in groups of always 2–3 cells in front of and behind the pigmented eyes (Figure 1D, E). These extraocular phaosomal PRCs are invisible in living animals. In juveniles these PRCs primarily form a pair of bands situated in the outer quarter of the animals (Figure 1D). The PRCs are located in the connective tissue underneath the epidermis (Figure 1D). In the juveniles studied with TEM up to seven phaosomes have been counted on a given cross section. The total number of extraocular PRCs has not been determined and appears to be related to the age of the individuals.

The extraocular PRCs are separated from one another by a thin layer of ECM (0.1-0.3 μm thick; Figures 1E and 2B). Thus, they are never in direct contact with one another. Each PRC houses a single, slightly ovoid phaosome approximately 6 by 6–8 μm in cross section and up to 10 μm in length (Figures 1E and 2B). The phaosome is located eccentrically in the cell body of each PRC (Figure 1E). The cell body contains plentiful mitochondria which are evenly distributed throughout the cell. Except for the region housing the nucleus, the cells form a thin cytoplasmic sheath of 0.3-0.6 μm around the phaosome (Figures 1E and 2B). The nuclei are somewhat flattened (2 × 5 × 5 μm) and contain a remarkably low amount of heterochromatin (Figure 1E). The phaosome is completely filled with densely packed sensory microvilli (Figure 1E). In most regions of the phaosome the microvilli are oriented strictly parallel; only in certain regions are the microvilli sectioned in different directions (Figure 1E, F). The microvilli are 2.5-3 μm long, have a diameter of 20–40 nm and are separated by a 10-nm-wide gap. Each microvillus contains a prominent bundle of thin filaments (Figure 1F).

Analyses of serial TEM sections through the brain region in *H. robusta* did not give any indication for the existence of ciliated sensory cells in the CNS.

### Bioinformatic analyses and amplification of genes

Bioinformatic analyses of the published genome of *H. robusta* resulted in sequence predictions of four *opsins* (in the following called *opsin A, B, C*, and *D*), two *G*_*αq*_*-subunits* of guanine nucleotide-binding proteins, one *tryptophane-2,3-dioxygenase* and one *sepiapterine reductase*. Despite thorough additional searches by means of a newly developed algorithm specifically for searches of opsin sequences, no additional *opsins* could be found. Based on these predictions, specific primers were designed and used for both *H. robusta* strains.

**Figure 3:**
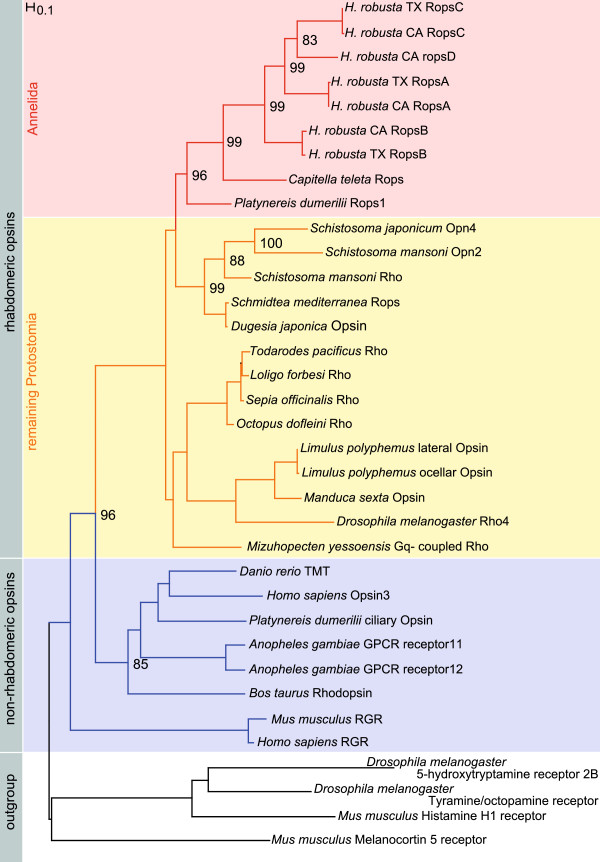
**Phylogenetic analysis of the cloned opsin sequences.** The *Helobdella* opsins cluster well supported (96%) within the rhabdomeric opsins. They form a clade with the r-opsins from *Platynereis dumerilii* and *Capitella teleta*. The different leech opsins are more closely related to each other than separated according to the genetic strain. The tree was calculated using RAxML with 1000 puzzling steps for the bootstrap values (given in percentage). Red: annelid rhabdomeric opsins. Yellow: remaining protostome rhabdomeric opsins. Blue: non-rhabdomeric opsin families. White: Outgroup.

*Opsins A, B* and *C* were amplified from cDNA of both strains. The predicted fourth *opsin D* could neither be amplified from cDNA nor from genomic DNA of either strain. The experimentally obtained sequences closely match the predictions and there are only insignificant differences on the nucleotide levels between the *opsins* of the two populations *H. robusta* CA and *H. robusta* TX. The phylogenetic tree in Figure 3 shows this as the opsins cluster according to their subtype A, B and C, and not according to the respective *Helobdella* strain. The phylogenetic analysis shows that all obtained opsins fall into the rhabdomeric opsin sub-tree and therein group together with the other known annelid rhabdomeric opsins. Within this cluster the opsins of *H. robusta* form one distinct and highly supported group (Figure 3). In this analysis the rhabdomeric opsin of *Capitella teleta* is sister to those of *H. robusta.*

**Figure 4:**
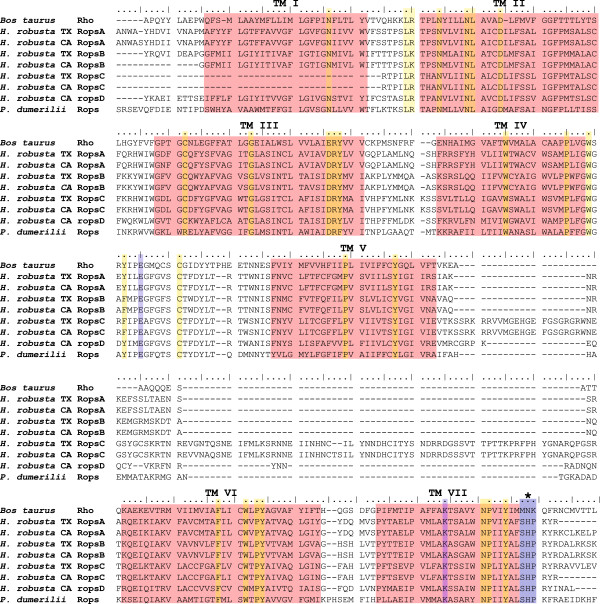
**Amino acid alignment of opsin sequences.** The four *H. robusta* opsins of each strain are compared to the ciliary rhodopsin from *Bos taurus* and the rhabdomeric opsin from *Platynereis dumerilii*. All opsins show seven transmembrane domains (TM I- VII) typical for G-protein coupled receptors, the protein family opsins belong to. Amino acids conserved throughout all opsins are highlighted in yellow. Amino acids essential for every functional visual opsin are marked in blue: The Glutamate (E) marked between transmembrane domain IV and V serves as counterion to stabilize the Schiffbase-linkage of the chromophore to the Lysine (K) located in domain VII. The motif SHP is marked with an asterisk which is distinctive for rhabdomeric opsins and distinguishes them from the ciliary opsins.

All four predicted opsins show the typical SHP amino acid motif following the seventh transmembrane domain, clearly identifying them as rhabdomeric opsins [8] (Figure 4). The alignment shows the seven transmembrane domains typical for G-Protein coupled receptors the protein family opsins belong to. The highly conserved Lysine residue (K296 in the bovine opsin) in the seventh transmembrane domain is essential for the Schiff-base linkage of the chromophore and identifies functional opsins. Another essential amino acid is the counter-ion Glutamate (E) in non-vertebrates at position 181 [11,26].

The specific primers for the *G*_*αq*_ subunits only worked for the strain *H. robusta* CA. Not even degenerate primers helped to amplify *G*_*αq*_ subunits for *H. robusta* TX. The genes *tryptophane-2,3-dioxygenase* and *sepiapterine reductase* were amplified from *H. robusta* TX in order to identify whether the pigments known to be employed in the eyes in other annelids are active in *H. robusta* as well [27].

### Gene expression analyses

Expression analyses of the *G*_*αq*_*subunits*, the *opsins* as well as *tryptophane-2,3-dioxygenase* and *sepiapterine reductase* were carried out by WMISH and additionally for the *opsins* by *in situ* hybridization of paraffin sections.

**Figure 5:**
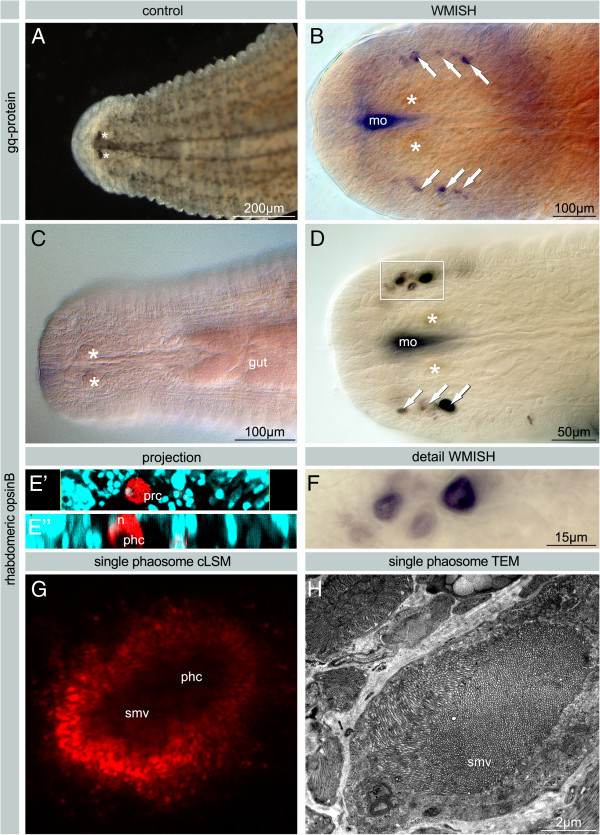
***H. robusta.*** Whole mount *in situ* hybridizations stained with NBT/BCIP. The staining in the pharynx is background due the influx of RNA probe into the mouth cavity. **A**. Live image of a *H. robusta* TX individual to show position of pigmented eyes. **B**. Expression of *gq-protein* in extraocular PRCs in *H. robusta* CA. **C**. Negative control. **D**. Expression of *H. robusta* TX *opsin B* in extraocular PRCs. **E’E”**. Vertical section of LSM stacks with reflection of the NBT/BCIP staining (red) and nuclei stained by DAPI (blue) to show that the expression is restricted to the cell body and present neither in the microvilli nor in the nucleus. **F**. Higher magnification of boxed area from D. Darker and lighter colours of the staining in adjacent phaosomal PRCs are caused by the focus levels. The phaosomal cavities are visible as lighter areas. **G**. Z-projection of cLSM stack showing one PRC. Note the dark phaosomal cavity without any signal. Reflection of the NBT/BCIP staining (red (blue). **H**. TEM micrograph of a similar single phaosomal PRC. The phaosomal cavity completely filled with sensory microvilli corresponding to the dark central space in G. - A-D, F Bright field images. E+G confocal laser scanning microscope images. arrows: extraocular phaosomal PRC; asterisks: pigmented eyes; psc pigmented supporting cell; mo mouth opening; phc phaosomal cavity; n nucleus; smv sensory microvilli.

**Figure 6:**
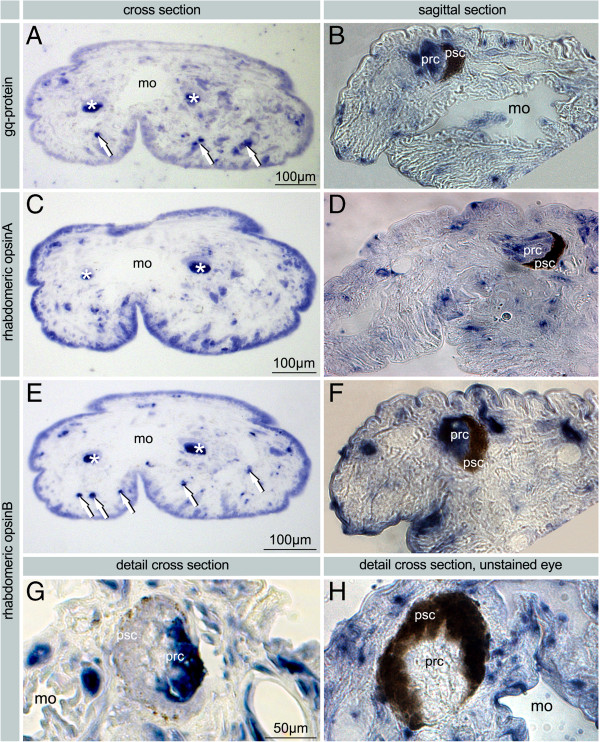
***In situ *on paraffin sections of *H. robusta *using NBT/BCIP.** Bright field images. **A**. Expression of *gq-protein* (CA) in the PRCs of the eyes (asterisks) and in the extraocular PRCs at the margin of the head (arrows). **B**. Expression of *gq-protein* (CA) in the PRCs of the eyes (prc) at higher magnification. **C**-**D**. Expression of *opsin A* (TX) in the PRCs of the eyes (asterisks, prc); and probably in the extraocular PRCs. **E**-**G**. Expression of *opsin B* (TX) in PRCs of the eyes (asterisks, prc) and in the extraocular PRCs (arrows). **F**. Expression signal in the eyes is restricted to the PRCs **G**. High magnification image with no expression in PSCs (psc) which lost pigment granules almost entirely during in situ hybridization. Staining of probe is restricted to cell bodies of PRCs, phaosomal cavities remain unstained. **H**. Sagittal section of unstained specimen showing pigmented eye with pigment granules and no staining in the PRCs. – arrows: extraocular phaosomal PRC; asterisks: pigmented eyes; mo mouth opening; phc phaosomal cavity; psc pigmented supporting cell; px pharynx; smv sensory microvilli.

One of the G_αq_ subunits is expressed in the extraocular phaosomes (Figures 5A, B and 6A, B). Expression of the second G_αq_ subunit could not be detected.

**Figure 7:**
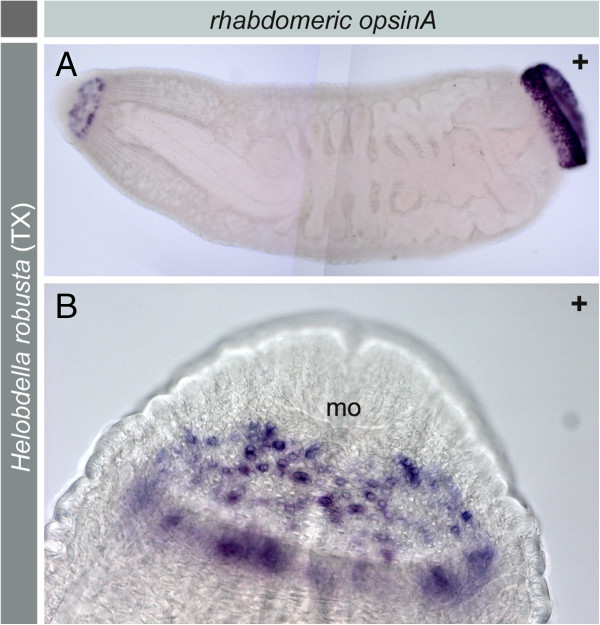
***H. robusta *TX.** Expression of *opsin A* in the brain region, anterior and posterior sucker. Whole mount *in situ* hybridizations of adult specimen stained with NBT/BCIP. **A**. Entire specimen showing a stronger signal in the posterior end. **B**. Close up of the anterior region showing that the signal is restricted to circularly arranged single cells. In each cell the signal is concentrated in the periphery.

Expression of *opsin A* in the pigmented eyes and in unpigmented single phaosomal PRCs could be shown in paraffin sections (Figure 6C, D). In whole mounts only the extraocular phaosomal PRCs are detectable (Figure 5D). As to be expected [24], in adults there is a strong ring-shaped expression in the region of the anterior and the posterior sucker due to the presence of phaosomal PRCs (Figure 7A). At higher magnification it is evident that it is caused by numerous single cells which are not arranged in a strictly bilateral symmetric pattern. In the anterior sucker there are at least 40 cells on each side of the body, which is by far exceeded in the posterior sucker (Figure 7B). *Opsin B* could be detected in the PRCs on sections and additionally in whole mounts, whereas the supportive pigment cells (PSCs) remained unstained (Figures 5A, C-G and 6F-G). Even after complete bleaching of the shading pigment there is no signal in the PSCs whereas there is a strong signal in the cell bodies of PRCs (Figure 6G). At higher magnification it becomes obvious that the staining is weaker in the centre of the PRC (Figure 5F). The LSM reflection shows that the messenger RNA of *opsin B* is located only in the cell body and not in the microvilli themselves (Figure 5G). That this region in fact represents the phaosomal cavity and not the unstained nucleus is shown in Figure 5E through an additional DAPI staining. The 3D projection of the LSM scans shows the blue-stained nucleus located next to the unstained phaosomal cavity (Figure 5E). Thus, the messenger is not stored in the microvilli themselves. This is in accordance with the appearance of the phaosomal PRCs in the TEM, clearly showing the phaosome surrounded by a sheath of cytoplasm and the nucleus being situated in a lateral extension of the cell body in the case of extraocular phaosomal receptor cells (Figures 1E and 5H). The same can be observed from the expression pattern in the extraocular phaosomes in the head region of adults (Figure 7B). The expression site of *opsin C* remains unknown as no *in situ* experiment showed positive results (data not shown).

**Figure 8:**
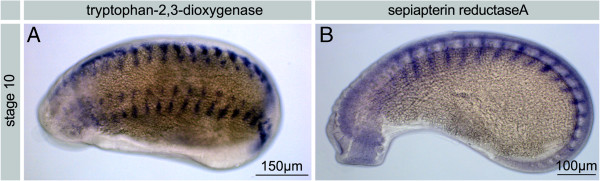
***H. robusta *TX.** Expression of pigment genes. Whole mount *in situ* hybridizations stained with NBT/BCIP, stage 10. **A**. Expression of *tryptophan-2,3-dioxygenase*. **B**. Expression of *sepiapterin reductase A*. For both genes a staining in the eye region is virtually absent.

Most likely SPCs do not express the pigment genes investigated: neither *tryptophane-2,3-dioxygenase* nor *sepiapterine reductase* expression was found to occur in either PSCs or PRCs. However, WMISH showed a segmental expression pattern in the epidermis demonstrating that the experimental protocol worked, at least in a subset of tissues (Figure 8A-B). This expression pattern observed is related to the developmental stages: in juveniles older than stage 11 (data on stage 11 not shown; staging according to [28]) the expression of both genes could no longer be detected. For the time being the pigment present in the PSCs remains unknown.

## Discussion

### Evolutionary origin of phaosomal PRCs

#### Morphological evidence

The main objective of the present investigation was to clarify the evolutionary origin of the phaosomal PRC typical of Clitellata. The overall uniformity in clitellate PRC structure is in contrast to the situation found in the other annelids, which exhibit a remarkable diversity in PRCs and eyes [13-15,29,30]. Whereas Jamieson [15,29] regarded the phaosomal PRCs as plesiomorphic mainly by inferring that simple equals primitive, Purschke [16] reached an opposite conclusion. He regarded these PRCs as being highly derived structures developed after the PRCs and eyes usually present in annelids had been lost, supposedly in connection with a change of life style in the stem lineage of Clitellata. It remained unresolved whether the third type of PRCs, the phaosomal PRC, evolved independently from rhabdomeric and ciliary PRCs or from one of the latter two [13,16].

The ultrastructure of clitellate phaosomes reveals that they primarily comprise microvilli (e. g., [17,20,31]). Together with similarities in the phototransduction mechanism [25] this seems to indicate a relationship to rhabdomeric PRCs. An origin from rhabdomeric annelid PRCs would raise the question whether the clitellate PRCs have evolved from larval or adult eyes, since a pair of pigmented larval eyes and at least one pair of adult eyes belong to the ground pattern of Annelida, and both of these employ rhabdomeric PRCs [2,13,32-34]. Moreover, the rhabdomeric PRCs present in the adult eye of *Capitella* spp. structurally resemble the phaosomal PRCs present in certain oligochaete clitellates such as *Stylaria lacustris* (Linnaeus, 1767) and *Eisenia fetida* (Savigny, 1826). In these annelid species these PRCs are still situated in the epithelial continuity of the epidermis and their phaosomes show a connection to the subcuticular extracellular space [16,18,35]. Such structural peculiarities are unknown in other polychaetes. Thus, likelihood appears to be greatest that *Helobdella* phaosomes are derived from adult rhabdomeric PRCs. These observations are in concordance with a recently published phylogeny of Annelida based on phylogenomic demonstration of Clitellata in a highly derived position [34,36].

#### Molecular and gene expression data

From our genomic studies it is evident that the opsins present in *H. robusta* belong to the rhabdomeric type. Although opsin sequences are currently available only from a few annelids, the phylogenetic analyses show that the opsins from *H. robusta* form one distinct and highly supported cluster within the annelid r-opsins, which in turn cluster as a single well supported group within protostomian r-opsins. Moreover, the *in situ* experiments show that two of the r-*opsins* are expressed in the phaosomal PRCs. Thus, phaosomes are derived from annelid rhabdomeric PRCs with a high degree of probability. The hypothesis that they represent a plesiomorphic type of PRC clearly has to be rejected [15,16,29]. The position of the *Helobdella* opsins in the phylogenetic tree suggests that their diversification most likely occurred after separation of Clitellata from the remaining annelids.

Whereas two out of four *opsins* in *H. robusta* are clearly related to photoreception, the function and expression site of the other two remain unclear. Among these, *opsin D* could not be cloned from cDNA and might therefore be expressed at another life history stage. *Opsin C* was cloned from cDNA but in situ expression analysis remained unsuccessful. The unusual large insert between the 5^th^ and 6^th^ transmembrane domain may indicate a divergent function for this opsin.

Whether the phaosomal PRCs evolved from larval or adult eyes cannot be answered from the opsins alone, since both types of eyes use the same set of r-opsins in *Platynereis dumerilii* (Audouin & Milne-Edwards, 1834). In *P. dumerilii* larval and adult eye PRCs may either be distinguished by their neurotransmitters, acetylcholine or glutamate, respectively, or by the presence of *Gq-alpha* in the PRCs of adult eyes [32,37]. Unfortunately, neurotransmitters have not been studied in *H. robusta* but the phaosomal PRCs express a Gq protein which is characteristic for annelid adult eye PRCs.

#### Evolutionary scenario of clitellate PRC evolution

Presuming that phaosomal PRCs originated from rhabdomeric PRCs, the question arises how and why phaosomes evolved. First, the development is direct inside a cocoon, so that the trochophore larva typical of annelids was probably lost [38-40]. Since this is one autapomorphic character of Clitellata, this loss must have happened in the clitellate stem lineage. With loss of the larva very likely its entire sensory equipment got lost as well.

Second, clitellates usually are infaunal, inhabiting various types of sediments and soils (e.g. [41]). In such environments vision is of minor importance and numerous examples for reductions of pigmented eyes exist throughout various metazoan lineages. Therefore, it seems conceivable that eyes typical of non-clitellate annelids were lost during invasion of deeper sediment zones.

This scenario applies for the PSCs but probably not for the rhabdomeric PRCs. The primary function of such PRCs might have been triggering the endogenous clock as well as basic detection of light resulting in light-avoiding reactions [21]. In other annelids the former functions are usually related to the cilary PRCs [7,8] which in Clitellata were lost as well, as indicated by the obvious lack of any *c-opsin* in the genome of *H. robusta*.

#### Cytological and structural constraints

Structurally photoreception is typically related to the apical membrane domain of epithelialized cells, i.e. to either cilia or microvilli projecting into an extracellular space. To provide as much membrane surface as possible for housing a high number of such cell processes only two possibilities exist: out-folding or infolding of the apical plasma membrane. The former usually is realized in most annelid eyes and this requires supporting cells to seal the extracellular space from the opposite side [13,14,33].

If supportive cells are absent, the only alternative for forming such an extracellular space is by infolding of the apical plasma membrane of the PRC and finally closure of this space, resulting in a seemingly intracellular vacuole, the phaosome. Interestingly, in the clitellates *Stylaria lacustris, Lumbriculus variegatus* and *Eisenia fetida* the phaosomes structurally represent such an intermediate stage in phaosome formation [16,18,21]. Completely closed phaosomal cavities are typical of leeches where the respective sensory cells are usually deeply embedded in the body tissues [22,31]. However, this feature is not restricted to this group and has been reported for the phaosomes of e.g. *Lumbricus terrestris* as well, indicating convergent events [19,20].

### Evolution of clitellate eyes

Eyes as generally present in leeches and their PSCs very likely evolved de novo in Clitellata. From the present results there are several indications for this hypothesis: (1) different pigment genes are involved in pigment formation in leech PSCs, (2) the PSCs are structurally separated by the ECM from the PRCs, and (3) within Clitellata pigmented eyes are only present in taxa having a comparatively derived phylogenetic position.

In *Platynereis dumerilii* both cell types forming the pigmented eye, PSCs and PRCs, express genes involved in the pathways of Pterin and Ommochrome synthesis [27,35,42]. We could not detect any signal of these pathways in either the eyes or the phaosomal PRCs in *H. robusta* although the genes are expressed in the trunk, which might serve as an indication for a lack of expression in the eyes. Loss of expression of these genes in older stages is consistent with observations in other animals.

As mentioned above, in Clitellata pigmented eyes are restricted to three taxa: Naidinae, Pristinae and Hirudinea. Both of these form high end terminals in the respective branches in the phylogenetic trees published [43-46]. This is indicative not only for an independent origin in Clitellata but likewise for a convergent evolution in the two groups as well; this most likely occurred together with an independent change to an epibenthic life style in the two groups [16].

### Leech PRC diversity

Leech eyes and extraocular phaosomes are well known from light microscopic observations since the end of the 19^th^ century [23,24,47]. Extraocular phaosomes have thus far been reported mainly from the region around the eyes, from segmental sensillae and from the posterior sucker where they in certain cases may even form simple eyes [23,24,47]. Electron microscopic observations have been carried out in *Helobdella stagnalis, Helobdella triserialis* (Blanchard, 1849)*, Haementeria depressa* Ringuelet, 1972, *Mooreobdella microstoma* (Moore, 1901) as well as in *Hirudo medicinalis* Linnaeus, 1758 (see [17,22,31,48]). In contrast to oligochaete clitellates leech phaosomes generally show little variation between species [19-21]. However, so far no ultrastructural data on extraocular phaosomes in leeches have been reported. From the present investigation it is obvious that in general the phaosomal PRCs show no differences, regardless of whether they are situated within or outside the eyes. In the eyes of all leech species studied the pigment cup is separated from their PRCs by a thin but conspicuous ECM. This feature at least seems to indicate that their precursor cells have been separated quite early in development and may even belong to different germ layers, i.e. ectoderm and mesoderm.

Unlike many other annelids ciliary PRC-like cells could not be detected either by screening series of ultrathin sections through the anterior region including the brain, or by screening the genome for a *ciliary opsin*. No ultrastructural data on ciliary PRCs have been reported elsewhere. This appears to be a good indication for the entire absence of such PRCs in Hirudinea. However, TEM investigations revealed a type of ciliary sense organ with presumed ciliary PRCs in certain microdrile oligochaetes [49-52]. Whether these ciliary sensors represent ciliary PRCs remains still to be proven. Several attempts failed to amplify and clone *c-opsins* with degenerated primers from the naidids *Pristina longiseta* (Ehrenberg, 1828), *Nais communis* (Piguet, 1906), *Tubifex tubifex* (Müller, 1774) as well as *Limnodrilus hoffmeisteri* Claparède, 1862. Thus, for the time being it can be assumed that in Clitellata PRCs are present only in the form of phaosomal rhabdomeric PRCs.

## Conclusions

The phaosomal PRCs in Clitellata very likely evolved from a precursor cell representing a rhabdomeric PRC as is present in pigmented eyes of various polychaetous annelids. Moreover, there is strong evidence that these PRCs represent the remnants of the adult polychaete eyes. Thus, phaosomes are a newly evolved and derived type of PRC rather than a primitive one. Since pigmented eyes in Clitellata are only present in two distinct and clearly separated terminal branches, convergent evolution of eyes is the most probable explanation. This scenario is congruent with the different modes of life style observed in Clitellata, which began with a change to an almost entirely endobenthic mode of life in the stem lineage of Clitellata [35,39,40,52]. During these changes most of the photoreceptive features typically present in polychaetes got lost except for a residual function of light detection in a specific cell type, the phaosomal PRC, taken from the rhabdomeric PRC present in the pigmented polychaete eyes. In two clitellate lineages, namely naidids and leeches, a second evolutionary trait led to colonization of epibenthic habitats and these lineages are characterized by possessing pigmented eyes which most likely evolved de novo in convergent evolutionary lines.

## Material and methods

### Material

The study was conducted on two laboratory strains of *Helobdella robusta* (Shankland et al., 1991); one from Austin, Texas, one from Sacramento, California. Both strains, abbreviated as *H. robusta* TX and *H. robusta* CA, were obtained from Prof. Weisblat, University of California, Berkley, USA (identifiers in [53]): TXAU-3 for the strain from Texas and CASA-4 for the strain from California). From the latter the genome has been sequenced [53] and for the former an in situ protocol was available. Animals were kept at room temperature in 1% artificial sea water and fed with freshwater snails. For experiments the anterior ends of juveniles which already had pigmented eye spots were used.

### Electron microscopy

For transmission electron microscopy juveniles at an age of more than eight days were chosen. Animals were relaxed in carbonated water prior to fixation. They were fixed in 2.5% glutaraldehyde in a 0.1 M phosphate buffer (pH 7.2) for 2.5 h at 4°C. After initial fixation, the anterior part was cut off and further processed. After rinsing in the same phosphate buffer (2.5 h, 7 changes, 4°C) specimens were post-fixed in a buffered 1% solution of OsO_4_ (1 h, 4°C). After a short wash with buffer (5 min) specimens were dehydrated in a graded ethanol series (starting with 30% and ending with 100% ethanol). Ethanol was successively replaced by the intermedium propylene oxide followed by a 1:3 mixture of the embedding medium with the intermedium. Propylene oxide was allowed to evaporate overnight and then final embedding took place in an Epon-Araldite mixture (polymerization at 60°C for 2 d). Ultrathin sections of the eye region from two specimens and the brain region of one specimen were obtained with a diamond knife (Diatome 45°) on Leica Ultracut E or Leica UCT ultramicrotomes. Ribbons of sections were collected on single slot grids coated with pioloform support films in order to obtain almost complete series of ultrathin sections. They were stained with 2% aqueous uranyl acetate for 40 minutes at 20°C and lead citrate (2.66 g Pb(NO_3_)_2_ + 3.52 g Na_3_C_6_H_5_O_7_ × 2 H_2_O in 100 ml H_2_O) for 6 minutes at 20°C in a Nanofilm Phoenix Ultrostainer. Finally the sections were examined using a Zeiss EM 902A electron microscope. Images were recorded digitally on a CCD camera.

### RNA extraction

Juvenile animals, which already had pigmented eye spots, were removed from the adults and starved for at least three days to avoid contamination with nucleic acids of the snails the leeches fed on. When there was no visible gut outline anymore the juveniles were transferred as dry as possible to 1.5 ml tubes which were immediately placed into liquid nitrogen for 5 to 10 minutes.

To homogenize the tissue a stone mortar and pestle, which were sterilized by 10 minutes incubation in 0.5 M sodium hydroxide, rinsed with ddH_2_O and autoclaved, were placed on dry ice. After the tools were chilled the frozen tissue was quickly ground to fine particles which were finally mixed with 1 ml peqGOLD Tri-Fast™ reagent (Peqlab Biotechnologie GmbH, Erlangen, Germany).

After successful homogenization of the tissue RNA was extracted according to the TRIzol® reagent protocol (Life Technologies, Inc., Gaitersburg, USA) with an addition of a high-salt precipitation solution (0.8 M sodium citrate, 1.2 M sodium chloride) during the RNA precipitation step to avoid contaminations with proteoglycan and polysaccharides. The quality of the extracted RNA was assessed using a NanoDrop ND-1000 spectrophotometer (Peqlab).

### cDNA cloning

For the generation of all complementary DNAs (cDNAs) the SMART™ RACE cDNA amplification kit (Clontech Laboratories, Inc., Mountain View, USA) was used. The recommended reverse transcriptase was substituted by SuperScript™ II RNase H- Reverse Transcriptase (Invitrogen Corporation, Carlsbad, USA). Sequence predictions for four *opsins* were found and Dr. Florian Raible, Max F. Perutz Laboratories, Vienna, provided predictions for two *g-q-alpha proteins* based upon the published genome of *Helobdella robusta* (CASA-4) [http://genome.jgi-psf.org/Helro1/Helro1.home.html]. The specific primers based on these predictions were always used to amplify the DNA fragments in both *H. robusta* strains. The following primer combinations were used: *H. robusta CA-RopsA*: CCCCATTGGTGGAAGTACCATGAC (forward), ATTAATTGTGCTTGTCAGCGCAATGG (reverse); *H. robusta TX-RopsA*: GATAGCGTCACGTGGTACAAAGAC (forward), ATTAATTGTGCTTGTCAGCGCAATGG (reverse); *H. robusta CA-RopsB*: GGTGGCTTCATGATAATCCTGGG (forward), TGTTGGGACCTCCTGAG (reverse);

*H. robusta TX-R*o*psB*: GGTGGCTTCATGATAATCCTGGG (forward), GATCCCTCCGTCTTCCACTGC (reverse); *H. robusta CA-RopsC:* CCAGGACTCCAATACTGCGAACCC (forward), CACCAACATCACTCCTCCTACTCC (reverse); *H. robusta TX-RopsC*: CCAGGACTCCAATACTGCGAACCC (forward), CACCAACATCACTCCTCCTACTCC (reverse); *H. robusta CA-gq*: TGGCGTGTTGTCTAAGTGAGGAG (forward), CGTCAAATAAAACATCCGTGTTC (reverse); *H. robusta TX-t23d*: CACGACGAACACCTCTTCATAGTTACTC (forward), TTACACAGCGCTTAATTCTGCAGTCAG (reverse); *H. robusta TX-sepr*: AATTGGTAGATGTTATTTCACCAC (forward), CATAGAAGAACGTTCTGTTTGATAACCG (reverse). The amplified PCR fragments were cloned into the plasmid pCRII®-TOPO® (Invitrogen) according to the protocol for One Shot® Chemical Transformation provided in the manual.

#### *In situ* hybridization

*Whole mount* in situ *hybridization (WMISH).* The linearization and transcription of the RNA probes was done for all samples according to the *Platynereis* standard protocol [54]. Following linearization of plasmids by restriction digestion using QIAquick® Nucleotide Removal Kit (Qiagen) the transcription was purified with the QIAGEN RNeasy® Mini Kit QIAGEN according to the RNA cleanup protocol. WMISH was carried out according to the protocol from Weisblat and Kuo [55]. As the specimens used were older juveniles and not embryos, the penetration of the probes was enhanced by using only the amputated anterior ends and an incubation for 30 min in 10 μg/ml ProteinaseK (from Tritirachium album; Merck) without shaking at room temperature. In addition a few similar WMISH experiments with adults were carried out as well.

Anti-acetylated α-Tubulin (Sigma, T7451, produced in mouse) was diluted 1:200, and the anti-DIG-Fab (Roche Deutschland Holding GmbH, Grenzach-Wyhlen, Germany) 1:4000 with blocking solution. The animals were incubated over night at 4°C followed by 1 hour at RT. The antibody solution was washed off with two rinses, 3 × 15 min, 3 × 1 hr in PTW. After the NBT/BCIP staining reaction the animals were blocked for 1hour at RT and then incubated over night at 4°C in Anti-Mouse FITC (diluted 1:125) and DAPI (1 μg/ml).

In situ *Hybridization on sections.* Paraffin embedding, sectioning and ISH on paraffin sections were carried out according to Cardona et al. [56]. Individuals of adult *H. robusta* (TX & AU) were fixed in 2%PFA/ 0.1% glutaraldehyde/ 1xPBS. Probe concentration was 2 ng/μl according to the standard leech protocol. The embedded animals were sectioned into 10 μm slices.

### Phylogenetic analyses of opsin sequences

*Sequence analyses.* The sequenced cDNA fragments were translated into Protein sequences and aligned with sequences obtained from NCBI using the in BioEdit incorporated ClustelW algorithm. The phylogenetic tree was calculated with RaXml using the protein model ‘LG’ obtained through ProtTest [57]. The substitution model was complemented with invariable substitutions and CAT based.

*Accession numbers for opsin sequences*. *H. robusta* CA RopsA [jgi|Helro1|85596 |e_gw1.47.45.1], *H. robusta* TX RopsA [KF613602], *H. robusta* CA RopsB [jgi|Helro1|132379|gw2.39.178.1], *H. robusta* TX RopsB [KF613603], *H. robusta* CA RopsC [jgi|Helro1|84106|e_gw1.39.176.1], *H. robusta* TX RopsC [KF613604], *H. robusta* CA RopsD [jgi|Helro1|129809|gw2.47.141.1], *Capitella teleta* Rops [jgi|Capca1|202516|fgenesh1_pg.C_scaffold_376000014], *Platynereis dumerillii* Rops1 [CAC86665.1], *Schistosoma mansoni* Rho [AAF73286.1], *Schistosoma mansoni* Opn2 [XP_002581174.1], *Schistosoma japonicum* Opn4 [CAX73070.1], *Dugesia japonica* [CAD13146.1], *Schmidtea mediterranea* Rops [AAD28720.1], *Todarodes pacificus* Rho [P31356.2], *Loligo forbesi* Rho [P24603.1], *Sepia officinalis* Rho [O16005.1], *Octopus dofleini* Rho [P09241.1], *Limulus polyphemus* ocellar opsin [P35361.1], *Limulus polyphemus* lateral opsin [P35360.1], *Drosophila melanogaster* Rho4 [AAA28856.1], *Manduca sexta* opsin [O02464.2], *Mizuhopecten yessoensis* Gq-coupled Rho [BAA22217], *Danio rerio* [AAL83431.1], Homo sapiens opsin3 [AAH36773.1], *Platynereis dumerillii* ciliary opsin [AAV63834.1], *Anopheles gambiae* GPCR receptor1 [AGAP002443-PA], *Anopheles gambiae* GPCR receptor2 [AGAP002444-PA], *Bos taurus* Rho [NP_001014890.1], *Homo sapiens* RGR [NP_001012740.1], *Mus musculus* RGR [NP_067315.1], *Drosophila melanogaster* 5HT-dro2B receptor [CAA77571.1], *Drosophila melanogaster* Tyramine/octopamine receptor [P22270.2], *Mus musculus* Histamine H1 receptor [NP_001239572.1], *Mus musculus* Melanocortin 5 receptor [AAI00721.1 GI: 71682941].

*Accession numbers for other sequences. H. robusta* CA tryptophan 2,3 dioxygenase [scaffold_18|1087627|1088299 (673 bp)], *H. robusta* TX t23d [KF613600], *H. robusta* CA sepiapterin reductase [scaffold_77|451749|452651 (903 bp)], *H. robusta* TX sptr [KF613601], *H. robusta* CA Gq [jgi|Helro1|184821|estExt_fgenesh4_kg.C_10016].

### Image Processing

Images were recorded digitally and further processed using Adobe Photoshop® and Adobe Illustrator®. 3D reconstructions (Figure 4E’, E”) were carried out by Raju Tomer, EMBL Heidelberg, with Imaris 5.7.1 (Bitplane AG, Zurich, Switzerland).

## Abbreviations

PSC: Pigmented supportive cell; PRC: Photoreceptor cell.

## Competing interests

The authors declare that they have no competing interests.

## Authors’ contributions

GP, DA and HH conceived the study. CD conducted most of the experimental work except for TEM and took the lead of data collection. JG carried out the TEM observations. KTR aided substantially in performing the molecular studies. HH and CD carried out the gene predictions. GP took the lead on writing the manuscript; GP and CD were the main contributors to the writing. HH, DA and KTR contributed substantially to the writing of the manuscript. All authors read and approved the final manuscript.
